# Antimicrobial Activity and Toxicity of Analogs of Wasp Venom EMP Peptides. Potential Influence of Oxidized Methionine

**DOI:** 10.3390/antibiotics10101208

**Published:** 2021-10-04

**Authors:** Roberto de la Salud Bea, Lily J. North, Sakura Horiuchi, Elaine R. Frawley, Qian Shen

**Affiliations:** 1 Department of Chemistry, Rhodes College, 2000 North Parkway, Memphis, TN 38112, USA; 2 Department of Chemistry, The University of Arizona, Tucson, AZ 85721, USA; lilyjnorth@email.arizona.edu; 3 School of Medicine and Health Sciences, George Washington University, 2300 I St., NW, Washington, DC 20052, USA; shoriuchi2@gwu.edu; 4 Department of Biology, Rhodes College, 2000 North Parkway, Memphis, TN 38112, USA; frawleye@rhodes.edu (E.R.F.); shenq@rhodes.edu (Q.S.)

**Keywords:** antimicrobial peptides, venom, wasp, methionine

## Abstract

The antibiotic and toxic properties for four synthetic analogs of eumenine mastoparan peptides (EMP) have been tested. These properties were compared to two natural peptides found in the venom of solitary wasps *Anterhynchium flavomarginatum micado* (natural peptide EMP-AF) and *Eumenes rubrofemoratus* (natural peptide EMP-ER), respectively. Only EMP-AF-OR showed concentration-dependent growth inhibition against all bacterial species tested. Gram positive species had MIC values of 10 μg/mL for *B. subtilis* and 25 μg/mL for *S. aureus.* Gram negative species had MIC values of 25 μg/mL for *E. coli* and 200 μg/mL for *P. aeruginosa*. Of the other tested peptides, EMP-ER-D2K2 also showed activity and inhibited growth of *Bacillus subtilis* in a concentration-dependent manner at 200 μg/mL. Peptide EMP-ER-OR reduced the final density of *Escherichia coli* and *B. subtilis* cultures but did not impact their growth kinetics. Peptides EMP-AF-OR, EMP-ER-OR, and EMP-ER-D2K2 showed limited antifungal activity against *Candida albicans* or *Histoplasma capsulatum*. The hemolytic activity of the analogs were moderated though reports of the natural peptides, especially EMP-AF-OR, already showed low toxicity against erythrocytes. These results are discussed in the context of the potential influence of oxidized methionine on EMP activity.

## 1. Introduction

One of the major current problems in medicine is the growing resistance of pathogenic organisms to traditional treatments. The situation is made worse by the abuse of antibiotics and the rapid selection of resistance variants [1,2,3]. It is therefore important to find new active compounds that not only have anti-microbial activity but also have mechanisms of action against which resistance is slower to evolve. Though combinatorial and parallel synthesis are the main techniques for the discovery of new drugs, there is a new interest in nature as the source of potential products with medicinal properties [4,5,6].

Venoms are natural products that some animals and plants use for defense against predators or, in the case of animals, to attack and catch prey. Venoms are complex mixtures of compounds. They may contain a variety of molecules from salts to amino acids to complex enzymes, which are toxic to those predators and prey. However, studies show that some components may have applications as potential drugs [7,8]. In fact, several toxins are currently used for the treatment of medical conditions. Captopril, a small molecule found in viper venom, is used to treat high blood pressure [9]. Ziconotide, a small peptide found in the venom of a marine cane snail, is used to treat severe pain [10]. Eptifibatide, a small cyclic peptide found in a rattlesnake venom, is used to prevent heart attacks and there are also studies reporting the use of venoms to treat cancer [11]. 

Antimicrobial peptides (AMP) are relatively short peptides containing between 5 to 100 amino acids in their sequence. They are found in nature as part of the immune defenses for plants and animals, including humans [12,13,14]. Despite their different origins, small peptides found in venoms have similar properties to AMP such as being amphipathic with cationic charges and with an alpha helix or beta sheet secondary structure [15]. Though there are several reported mechanisms of action for AMP, most of them are based on their interaction with and distortion of cell membranes [16]. Small peptides found in the complex mixture of compounds in the venom of snakes, frogs, insects, scorpions or spiders have been shown to function similarly to AMP. There are a variety of studies showing antibacterial, antifungal and even antitumor activity of these natural peptides as well as of synthetic derivatives or analogs [17,18].

There are thousands of species of wasps found around the world. The females have a stinger that injects venom and, unlike bees, wasps do not leave the stinger embedded in the prey and die after they sting. Therefore, wasps can use their venom throughout their lifetime. (https://www.britannica.com/animal/wasp, 24 April 2021). Wasp venom contains a library of components including small molecules that were identified as amino acids, biogenic amines and nucleic acids [19]. They also contain peptides with antimicrobial properties but, as expected from a venom, many of these peptides are also toxic to human cells, producing hemolytic activities among other undesired effects [20]. Mastoparans are a group of amphiphilic peptides that are 14 amino acids long, with amidated C-termini, found in the venoms of a variety of social wasps [21]. Though toxic, or perhaps because of their toxicity, studies have shown mastoparans with potential therapeutic applications [22,23]. Mastoparan-like peptides have now been discovered in the venoms of solitary wasps as well [24]. EMP-AF from *Eumenine Anterhynchium flavomarginatum micado* was the first to be purified and studied. This well-studied peptide has shown good antimicrobial activity and relatively low hemolytic activity. It is therefore of interest as a base from which to design effective antimicrobials. Two natural truncated analogs of EMP-AF were also purified and they showed no antimicrobial activity [25]. The second mastoparan-like peptide discovered, EMP-OD extracted from *Orancistrocerus drewseni,* had a different sequence than EMP-AF but also had lower antimicrobial activity and produced greater hemolysis [26]. The remaining mastaparan-like peptides that have since been identified from the venoms of different solitary wasp species have amino acid sequences that are similar to one another yet different from EMP-AF. However, like EMP-AF, they display desirable biological activities [24].

In this study, our goal was to design and synthesize a group of analogs of mastoparan-like peptides to determine whether altering net charge, orientation of amphiphilic sides, or peptide length would affect peptide structure or enhance the antimicrobial activities of the natural peptides while simultaneously reducing toxicity. We selected the original and best studied mastoparan-like peptide EMP-AF and a representative from the group of other similar mastoparan-like peptides, EMP-ER from *Eumenes rubrofemoratus,* as the model natural peptides from which analogs would be designed and synthesized.

## 2. Results

### 2.1. Peptide Design

The design of the analogs was based on the sequences of two natural peptides found in the venom of two wasps. From the wasp *Eumenine Anterhynchium flavomarginatum micado* we used the venom peptide EMP-AF (I N L L K I A K G I I K S L-NH_2_) to design the analog EMP-AF-KV (Table 1). From the wasp *Eumenes rubrofemoratus* we used the venom peptide EMP-ER (F D I M G L I K K V A G A L-NH_2_) to design three analogs: EMP-ER-KE, EMP-ER-D2K2 and EMP-ER-C3 (Table 1).

A total of six peptides were synthesized including the two natural sequences, called here EMP-AF-OR and EMP-ER-OR, and four analogs. Five of the analogs have 14 amino acids like the natural peptides while one, (EMP-ER-C3) has 11 amino acids. None have a disulfide bond. All peptides have a free N-terminus and an amidated C-terminus (Table 1). To test the influence of charges and hydrophobicity on the structure and activity of the analogs, specific positions on the hydrophobic or the hydrophilic side of the helix were systematically substituted with the hydrophilic amino acid lysine and one of them with glutamic acid. One analog has three amino acids removed from the C-terminus to test if this structural modification produces a significant difference in activity, either greater or reduced, from the longer ones [27], an idea Konno also suggested for EMP peptides [28]. According to the mass spectrometry results (Table 1), EMP-ER peptides show molecular masses 16 Da higher than the calculated masses which is due to the presence of methionine oxide. This fact does not affect the structural properties of these peptides such as alpha helical structure (Table 2).

**Figure 1:**
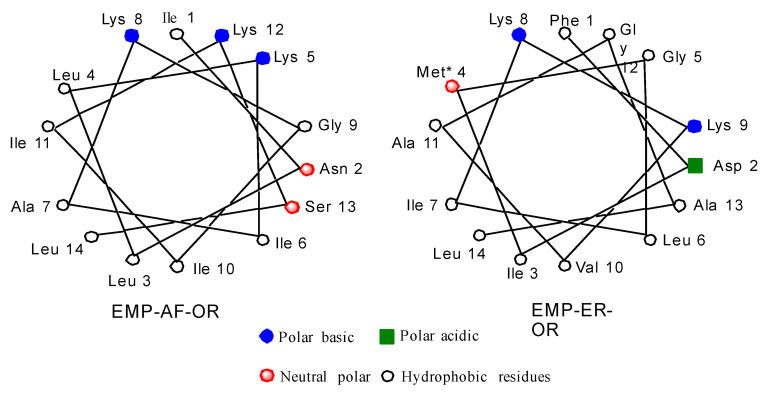
Schiffer and Edmunson alpha helix wheel projection of EMP-AF-OR and EMP-ER-OR peptides.

Schiffer and Edmunson projections for EMP-AF-OR and EMP-ER-OR natural peptides can be seen in Figure 1. Based on the model for the alpha helix structure for these peptides, it can be seen in the star projection for EMP-AF-OR that positions 5, 8 and 12 are on the charged side of the helix (Figure 1). In modified peptide EMP-AF-KV, the charged lysines at positions 5, 8 and 12 were substituted with hydrophobic amino acid valine while positions 3, 7 and 10 on the original hydrophobic side have been replaced with charged lysines, reversing the polarity of the whole peptide (Supplementary Materials Figure S1). For EMP-ER D2K2 the negatively charged aspartate from the original peptide was replaced with a lysine, increasing the net positive charge from +2 to +4. For EMP-ER KE, the opposite approach was used and the lysines were replaced with glutamates resulting in an analog with a net charge of −2. The EMP-ER-C3 peptide with the C-terminus truncated still retains the natural +2 net charge. The original natural peptides were also synthesized for reference and comparison.

### 2.2. Secondary Structure

All the peptides show some, though low, amounts of helical structuring in water except for EMP-ER-KE (Table 2). All peptides show a helical structure in the hydrophobic environment 50% trifluoroethanol (TFE) in water, especially the natural sequence EMP-AF-OR which seems to have fully helical structure (Table 2). This seems to be something intrinsic to this peptide since it also shows a higher ellipticity even in water. Curiously, the other peptide with a high percentage of helical structure is EMP-ER-C3, which is three amino acids shorter at the C-terminus and has only a +2 net charge. The rest of the analogs have a similar range of helical percentage (34% to 37%) and as mentioned above, the presence of methionine oxide seems not to make any difference if we compare to EMP-AF-KV, which does not have that amino acid. In the case of analog EMP-ER-KE, which has a net charge of −2, substitution of glutamic acid for the original lysine does not affect the helical structure in TFE, though it is the only peptide with a random structure in water (Table 2, Figure S2, Table S1).

### 2.3. Antibacterial Activities

The antibacterial activity of the natural peptides and analogs was determined using the standard microbroth dilution method for determining minimal inhibitory concentration (MIC) [29]. Only the original (natural) EMP-AF-OR peptide shows a significant antimicrobial activity against the tested bacteria with MICs ranging from 10 μg/mL for Bacillus subtilis, a Gram-positive bacterium, to 200 μg/mL for Pseudomonas aeruginosa, a Gram negative bacterium (Table 3, Figure S3). The analog EMP-ER-D2K2 is the only analog that has some, but low, activity against B. subtilis with an MIC of 200 μg/mL. Other peptides were able to suppress final culture densities but did not affect growth kinetics and no MIC could be determined (Figure S3).

### 2.4. Antifungal Activities

The antifungal activity of antimicrobial peptides was determined by a microplate-based growth assay. Amphotericin B was used as a positive control. The MICs of amphotericin B for all fungi tested in this study, except for *Aspergillus nidulans*, were in the range of 0.25–1.0 µg/mL (Table 4, Figure S4A), which is consistent with other published results [30,31,32]. EMP-AV-KV, EMP-ER-KE, and EMP-ER-C3 showed no activity against any of the fungi tested in this study as all MICs were greater than 256 µg/mL (Table 4), the highest concentration used for all peptides. EMP-AF-OR, EMP-ER-OR, and EMP-ER-D2K2 exhibited limited antifungal activities against two pathogenic fungi, *Histoplasma capsulatum* and *Candida albicans*. EMP-AF-OR was active against *H. capsulatum* with a MIC of 128 µg/mL (Table 4, Figure S4B). EMP-ER-OR and EMP-ER-D2K2 were active against *C. albicans* with both MICs of 256 µg/mL (Table 4, Figure S4C,D).

### 2.5. Hemolytic Activity

**Figure 2:**
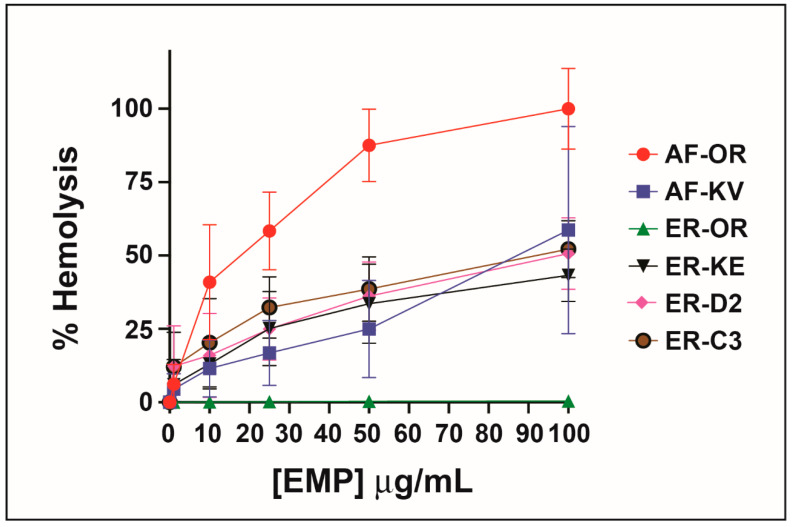
Hemolytic activity of peptide analogs.

When Konno first studied the biological properties of the peptide EMP-AF he reported induced hemolysis in human erythrocytes but he found a potency one-third of mastoparan though he shows a 75% release at concentrations of 0.5 μg/m. Other synthetic analogs of EMP-AF showed only weak hemolytic activity [28]. In our study, the original peptide (called here EMP-AF-OR) shows the highest hemolytic activity with 100% hemolysis at 0.1 μg/mL. For EMP-ER, Konno reports 100% hemolysis for a concentration of 0.5 μg/mL and about 50% for 0.18 μg/mL [33]. In our test, we found a similar activity for EMP-ER-OR and the studied peptide analogs against erythrocytes under the conditions and concentrations tested and there is a clear trend of increasing hemolysis with increasing concentrations. (Figure 2)

## 3. Discussion

The first biological studies carried out by Konno with the EMP-AF natural peptide show that it has common activities to mastoparans (MPs): they have amphiphilic alpha-helix conformations, and they have mast cell degranulating activity, but EMP-AF has about one-third the hemolysis potency of MPs. However, some analogs showed much lower activity, especially if their structures were truncated. Later studies showed that EMP-AF has a broad spectrum inhibitory activity against Gram-positive and Gram-negative bacteria [25]. Similar studies for the EMP-ER natural peptide showed that it also has potent antibacterial and moderate hemolytic activities [33].

Our results agree with the more potent antibacterial properties for EMP-AF (called in our work EMP-AF-OR). Interestingly, the analog EMP-AF-KV has no antibacterial activity, despite the fact that it has the same net charge as the natural peptide (+4) and it was designed to have switched their amphiphilic sides as shown on the helix projection (Figure S2). In the case of the natural EMP-ER peptide, it has been reported to have antibacterial activity [33], but our methionine oxidized EMP-ER-OR peptide and its analogs show no antimicrobial activity at the tested concentrations. 

Though there is a report of anti-yeast studies of these natural peptides [33], as far as we know, there are no other previous antifungal studies with them and their derivatives here tested. In that report EMP-ER showed anti-yeast activity. Our studies show no activity or limited activity against fungi tested in this work for any of the peptides, including EMP-AF-OR, which, as mentioned, does show antibacterial activity. This could be partly due to the distinct chemical composition of fungal cell wall. The presence of teichoic acid in the Gram-positive bacterial cell wall or lipopolysaccharides in the Gram-negative bacterial outer membrane confers negative surface charge which allows the electrostatic attraction with cationic antimicrobial peptides [34,35]. This initial interaction further facilitates the interaction between the bacterial plasma membrane and antimicrobial peptides, resulting in antibacterial activity by membrane distortion. The fungal cell walls is largely made of polysaccharides that are electrically neutral [36], thus the initial interaction between antimicrobial peptides with the fungal cell wall is less efficient compared to that in bacteria. In addition, fungi are eukaryotic organisms, thus compared to bacterial plasma membrane, their plasma membrane shares more similarity with the plasma membrane of mammalian cells which is relative resistant to antimicrobial peptides due to the lack of negatively charged phopholipids (e.g., phophatidylglycerol and cardiolipin) on the outer leaflet of the plasma membrane [37,38]. Therefore, even if antimicrobial peptides manage to interact with the fungal cell wall and have access to the fungal plasma membrane, the ability to distort the fungal plasma membrane and lyse the fungal cells will be limited. 

These limited antifungal and hemolytic activities also highlight that these analog peptides are not toxic to eukaryotic cells in general, except for the original peptide EMP-AF-OR. However, they also have low or no antibacterial activity. One possible explanation for these results is the fact that our synthetic EMP-ER peptides, based on our ES mass spectroscopy data, may have an oxidized methionine (sulfoxide). EMP-AF-OR and EMP-AF-KV do not have methionine in their sequence. There are reports that show oxidation of methionine to sulfoxides can activate or deactivate protein and enzyme activities but in these examples the oxidation either happens in binding or active sites, or it affects the conformation of tertiary and quaternary structures of such biological molecules [39,40]. In addition to impacting tertiary and quaternary structure of enzymes, methionine oxidation can create changes in the secondary structures of methionine-rich peptides from alpha helix to beta sheet [39,41]. The low antimicrobial activities observed for our AMP analogs were unexpected since, despite the oxidized methionine, our results show that these peptides have all the necessary structural features to be antimicrobial peptides: they form an alpha helix (not beta sheet) structure even in water (Table 2), they are amphiphilic as indicated by the helix projections of their alpha helix secondary structure (Figure S1) and also have a range of net charges from +4 to −2. Therefore, it is quite strange that even for the natural EMP-ER-OR peptide, a simple oxidation of methionine can change completely its antimicrobial activities from the reported non-oxidized one. A further study is in progress to compare these results with non-oxidized analogs.

## 4. Materials and Methods

### 4.1. Reagents

All Fmoc-protected amino acids, the activating reagent 2-(1H-benzotriazol-1-yl)-1,1,3,3-tetramethyluronium hexafluorophosphate (HBTU), and Rink-Amide AM Resin (200–400 nm mesh) used for the manual solid phase peptide synthesis were purchased from Nova-Biochem (EMD Group of Merk-Millipore KGaA, Darmstadt, Germany). Dimethyl formamide (DMF), acetonitrile, diisopropyl ethyl amine (DIPEA), piperidine, trifluoro acetic acid (TFA), bacteria and fungi broth were purchased from Fisher Scientific (Thermo Fisher Scientific, Waltham, MA, USA) to the purest quality and used without further purification. A fritted filter funnel (peptide reaction chamber, Reference Z284734-1EA) was from Sigma-Aldrich (St. Louis, MO, USA).

### 4.2. Peptide Synthesis and Purification

The synthesis of the peptides was carried out as previously described in our group [42] by manual solid-phase peptide synthesis using fluorenyloxycarbonyl (Fmoc) chemistry and 4-(2′,4′-dimethoxyphenyl-aminoethyl)-phenoxyacetamido-norleucylaminomethyl resin (0.4–0.8 mmol/g). Briefly, a solution of Fmoc protected amino acid, HBTU and DIPEA was added to a peptide fritted filter funnel containing a suspension of NH2 free Rink Amide resing pellets in DMF. The mixture was shaken for 30 min. After this time the liquid solution was filtered under vacuum. After pellets dried, a 20% solution of piperidine in DMF were added to the pellets and shaken for a total of 13 min, then filtered. The process is repeated for each amino acid until completion of the peptide sequence. The peptide was cleaved and fully deprotected from the resing by treating the pellets with a 95% solution of TFA in water and shaken for 1 h. The TFA solution containing the free peptide was evaporated under vacuum and the peptide precipitated as solid from addition of cold diethyl ether to the resulting oil.

Purification was carried out by reversed-phase high-performance liquid chromatography (RP-HPLC) with a water/acetonitrile gradient with a semipreparative C18 column at 40 °C. Peptides were characterized by mass spectrometry (LTQ-XL mass spectrometer positive mode of the ESI source). All peptides were dissolved in water or indicated buffers for each test to a concentration of 2 mg/mL to form standard solutions. The concentrations were determined by measuring the absorbance of the solutions at 220 nm (Peptide bond ε = 7000 cm^−1^ M^−1^) and 256 nm (Phe ε = 200 cm^−1^ M^−1^) and using the Lambert-Beer equation and molecular weights for each peptide.

### 4.3. Characterization of Helical Structure

The mean residue ellipticities of the peptides were determined by circular dichroism (CD) spectroscopy with an Aviv 401 spectrometer (Aviv Biomedical, Lakewood, NJ, USA) at 25 °C in water and in the presence of α-helix inducing solvent, 2,2,2-trifluoroethanol (TFE) at 50% in water. The concentration of peptides was 100 μg/mL for all the solutions, and they were loaded into a cell of 1 mm thickness. The scan range of wavelengths was from 250 to 190 nm at 1 nm intervals. The CD spectra were measured by averaging three scans. The mean residue molar ellipticity, [θ], is given in deg · cm^2^ · dmol^−1^and was calculated as [θ] = (θ × 0.1 × MRW)/(L × C), where θ is the experimental ellipticity, MRW is the mean residue weight (in Dalton), L is the light pathlength (in cm) and C is the concentration (in mg/cm^3^). Percentage helical content of the peptide was calculated as % helix = ([θ]_222_–[θ]${}_{222}^{0}$)/[θ${}_{222}^{100}$] × 100, where [θ]_222_ is the experimentally observed absolute mean residue ellipticity at 222 nm. Values taken for 0% and 100% helix content were [θ]${}_{222}^{0}$ = −2340 and [θ]${}_{222}^{100}$ = −30,300 respectively [43]. 

### 4.4. Bacteria Strains

*Escherichia coli* ATCC 25922 was obtained from ATCC and *Pseudomonas aeruginosa* ATCC 27853 was obtained from Carolina Biologicals. *Staphylococcus aureus* Newman was the *S. aureus* strain and the *B. subtilis* strain was JH642 (*trpC2, pheA1*) [44]. 

### 4.5. Measurement of Antibacterial Activity

Bacteria were grown on LB-agar plates (Fisher, Waltham, MA, USA) overnight at 37 °C, then stored at 4 °C until use. Four colonies were inoculated from the plate into 5 mL cation-adjusted Mueller-Hinton medium (Sigma) and grown to approximately OD_600_ = 1–2 at 37 °C in a shaking incubator at 250 rpm in preparation for antimicrobial activity testing. MICs were determined in cation-adjusted Mueller-Hinton broth using the microbroth dilution method from the Clinical and Laboratory Standards Institute [29]. Growth was monitored by determining the optical density at 600 nm using a BioTek Synergy HTX multi-mode 96-well plate reader set to incubate at 37 °C and record every 15 min for 20 h. Each assay was performed in biological triplicate.

### 4.6. Fungi Strains

*Saccharomyces cerevisiae* S288C, *Schizosaccharomyces pombe* 972h-, and *Aspergillus nidulans* FGSC A4 were kind gifts from Mary Miller, Bayly Wheeler, and Terry Hill, respectively at Rhodes College. *Candida albicans* SC5314 and *Histoplasma capsulatum* G217B were kind gifts from Chad Rappleye at Ohio State University. *S. cerevisiae* S288C, *S. pombe* 972h-, *A. nidulans* FGSC A4, and *C. albicans* SC5314 were maintained on yeast peptone dextrose (YPD) agar. *Histoplasma capsulatum* G217B was maintained on *Histoplasma*-macrophage medium (HMM) agar.

### 4.7. Measurement of Antifungal Activity

Fungal growth was determined over a range of individual peptide or amphotericin B concentrations. Each individual peptide and amphotericin B were solubilized in water at 512 µg/mL and 32 µg/mL, respectively. All samples were sterilized by filtration using 0.22 µm membranes. A 2-fold serial dilution was performed on each individual peptide or amphotericin B to obtain a concentration gradient. All fungal cultures were freshly prepared prior to the fungal growth assay. *S. cerevisiae* S288C, *S. pombe* 972h-, and *C. albicans* SC5314 were inoculated into YPD liquid medium and grown overnight at 30 °C with continuous shaking (200 rpm). About 2 × 10^5^ conidia of *A. nidulans* FGSC A4 were spread plated on a YPD agar. Fresh conidia were harvested after 48 h of static incubation at 30 °C. *H. capsulatum* G217B were inoculated into HMM liquid medium and grown for 48 h at 37 °C with continuous shaking (200 rpm). All fungal cultures were diluted and the cell concentrations were determined using a hemocytometer. For the fungal growth assay, *S. cerevisiae* S288C, *S. pombe* 972h-, *C. albicans* SC5314, and *A. nidulans* FGSC A4 were diluted to 2 × 10^4^ yeasts or conidia/mL in 2 × YPD liquid medium, and *H. capsulatum* G217B was diluted to 2 × 10^6^ yeasts/mL in 2 × HMM liquid medium. Fifty µL of each diluted fungal culture was mixed with 50 µL of individual peptide or amphotericin B with different concentrations in a 96-well plate. The highest concentration of each individual peptide and amphotericin B was 256 µg/mL and 16 µg/mL, respectively. Thereafter, *S. cerevisiae* S288C and *S. pombe* 972h- were incubated at 30 °C for 24 h. *C. albicans* SC5314 and *A. nidulans* FGSC A4 were incubated at 37 °C for 24 h. *H. capsulatum* G217B was incubated at 37 °C for 96 h. In the end of incubation, except for *A. nidulans* FGSC A4, all fungal growth was measured by optical density at 595 nm. Minimum inhibitory concentrations (MICs) were recorded. Due to the hyphae formation of *A. nidulans* FGSC A4, its MICs were recorded by visual observation after 24 h of incubation [31].

### 4.8. Measurement of Hemolytic Activity

Type O+ fresh human blood was purchased from Tennessee Blood Services Corp. (Memphis, TN, USA) in 10% citrate phosphate dextrose 10X phosphate saline solution (PBS) and EDTA (K2). Upon receiving the blood, it was washed using three volumes of 1X PBS + 5 mM glucose. The suspension was centrifuged at 1000× *g* for 10 min at 4 °C and the supernatant removed. The process was repeated two more times. The sedimented cells were resuspended in the same volume of 1X PBS + 5 mM glucose and stored at 4 °C until use.

Hemolytic activity was measured as follows: In 1 mL plastic tubes, serial concentrations of peptides (final concentrations of 0 as blank, 1, 10, 25, 50 and 100 μg/mL) were added to 1% human erythrocytes in 1X PBS in a total volume of 100 μL buffer. The cell suspensions were incubated at 37 °C for 1 h. The unlysed erythrocytes were removed by centrifugation (16,000× *g*) and the released hemoglobin was determined spectrophotometrically at 416 nm using a NanoDrop spectrophotometer (Thermo Fisher Scientific, Waltham, MA, USA). The control for no release of hemoglobin was a sample of 1% erythrocytes incubated in PBS without peptide. The control for 100% release of hemoglobin was a sample of 1% erythrocyte incubated in PBS with 1% TritonX-100. Each assay was performed in biological triplicate [45].

## 5. Conclusions

The antimicrobial, antifungal and hemolytic properties of two natural peptides and four analogs of antimicrobial peptides from wasp venom were studied. The antifungal properties are reported for the first time for these EMP wasp venom peptides. The results show that, though it does not have antifungal activity, the natural peptide EMP-AF-OR has the high antibacterial and toxic properties reported previously, but for the rest of the peptides antimicrobial activity is low. This is an unexpected result since all the analogs were designed with similar characteristics of any antimicrobial peptide, that is, they all show an alpha helical secondary structure, they all have amphiphilic structure and all have a net positive charge that ranges from +2 to +4, except one with a charge of −2. The fact that four of them may have oxidized methionine does not completely explain these results since this oxidation seems not to affect the basic structural requirements of antimicrobial peptides. This is quite interesting and we are working on further studies to characterize the critical chemical features of mastoparan-like EMP peptides and investigate the potential impacts of oxidized methionine on antimicrobial activity.

## Figures and Tables

**Table 1:** Amino acid sequences, Molecular Masses, HPLC Retention Time and Charges of EMP Peptides.

Peptide	Amino Acid Sequence	Calculated Molecular Mass (Da)	Observed Molecular Mass (Da)	HPLC Retention Time (min)	Charge at pH = 7
EMP-AF-OR	I N L L K I A K G I I K S L-NH_2_	1522.96	1522.96	21.6	+4
EMP-AF-KV	I N **K** L **V** I **KV** G **K** I **V** S L-NH_2_	1522.96	1522.96	16.8	+4
EMP-ER-OR	F D I M* G L I K K V A G A L-NH_2_	1474.87	1490.75	19.7	+2
EMP-ER-KE	F D I M* G L I E E V A G A L-NH_2_	1487.95	1503.90	24.5	−2
EMP-ER-D2K2	F **K** I M* G L I K K V A G A L-NH_2_	1476.75	1493.80	18.4	+4
EMP-ER-C3	F D I M* G L I K K V A-NH_2_	1233.58	1249.80	17.5	+2

Bold letters indicate the substituted amino acids. Asterisks (*) on Methionine indicate it is the sulfoxide form.

**Table 2:** α-Helical Content of EMP Peptides in Water and 50% TFE in Water.

Peptide	Water		50% TFE	
	[θ]_222_	% Helix	[θ]_222_	% Helix
EMP-AF-OR	−7711.94	17.73	−47,630.55	100
EMP-AF-KV	−4719.43	7.85	−13,692.93	37.47
EMP-ER-OR	−2759.13	1.38	−13,698.05	37.49
EMP-ER-KE	−2256.20	Random	−12,749.65	34.35
EMP-ER-D2K2	−240.03	6.93	−13,694.72	37.47
EMP-ER-C3	−109.85	7.36	−18,019.75	51.74

**Table 3:** MIC (μg/mL). Antibacterial activity of EMP analogs.

Peptide	MIC (μg/mL)			
	*S. aureus*	*B. subtilis*	*P. aeruginosa*	*E. coli*
EMP-AF-OR	25 μg/mL	10 μg/mL	200 μg/mL	25 μg/mL
EMP-AF-KV	ND	ND	ND	ND
EMP-ER-OR	ND	ND	ND	ND
EMP-ER-KE	ND	ND	ND	ND
EMP-ER-D2K2	ND	200 μg/mL	ND	ND
EMP-ER-C3	ND	ND	ND	ND

ND = not determined.

**Table 4:** Antifungal activity of EMP analogs.

Peptides	MIC (µg/mL)				
	*Sc*	*Sp*	*An*	*Ca*	*Hc*
EMP-AF-OR	>256	>256	>256	>256	128
EMP-AF-KV	>256	>256	>256	>256	>256
EMP-ER-OR	>256	>256	>256	256	>256
EMP-ER-KE	>256	>256	>256	>256	>256
EMP-ER-D2K2	>256	>256	>256	256	>256
EMP-ER-C3	>256	>256	>256	>256	>256
Amphotericin B	1.0	0.25	> 16	0.5	0.5

*Sc: Saccharomyces cerevisiae* S288C; *Sp: Schizosaccharomyces pombe* 972h-; *An: Aspergillus nidulans* FGSC A4; *Ca: Candida albicans* SC5314; *Hc: Histoplasma capsulatum* G217B.

## Data Availability

Data is contained within the article or supplementary material.

## Supplementary Materials

The following are available online at https://www.mdpi.com/article/10.3390/antibiotics10101208/s1, Figure S1: Schiffer and Edmunson alpha helix wheel projection of EMP-AF and EMP-ER peptides in this work, Figure S2: Circular Dichroism plots for secondary structure of EMP peptides, Figure S3: Antibacterial Activity experimental results: plots; Figure S4: Antifungal dose-response curves tested with 96-well microplate growth assay, Table S1: α-Helical Content of EMP Peptides in Water and 50% TFE in Water with errors.
