# Supplementary material for: Antimicrobial Activity and Toxicity of Analogs of Wasp Venom EMP Peptides. Potential Influence of Oxidized Methionine
Source: Antibiotics (Basel). 2021 Oct 4;10(10):1208. doi: 10.3390/antibiotics10101208 (PMC8532962; doi:10.3390/antibiotics10101208)
Supplement: Supplementary file 1 [file antibiotics-10-01208-s001.zip › antibiotics-1373660-Supplementary.pdf]

Supplementary Materials

Antimicrobial Activity and Toxicity of Analogs of Wasp Venom EMP Peptides. Potential Influence of Oxidized Methionine

Roberto de la Salud Bea, Lily J. North, Sakura Horiuchi, Elaine R. Frawley and Qian Shen

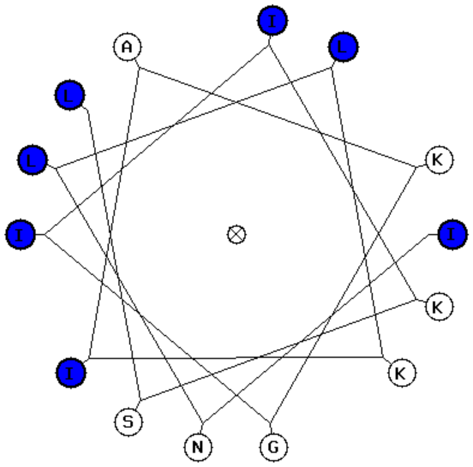

EMP-AF-OR

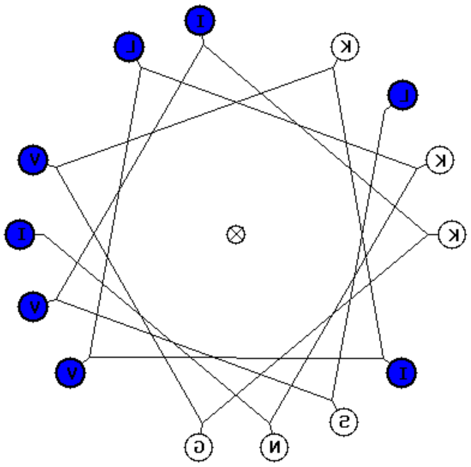

EMP-AF-KV

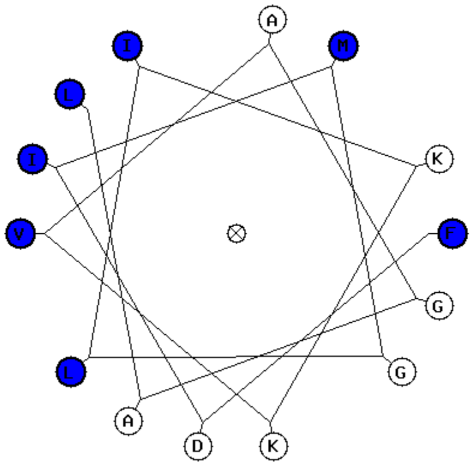

EMP-ER-OR

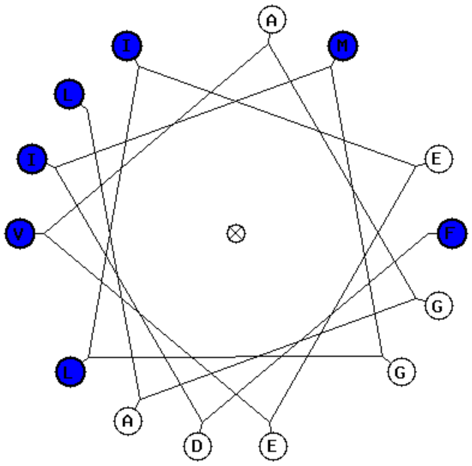

EMP-ER-KE

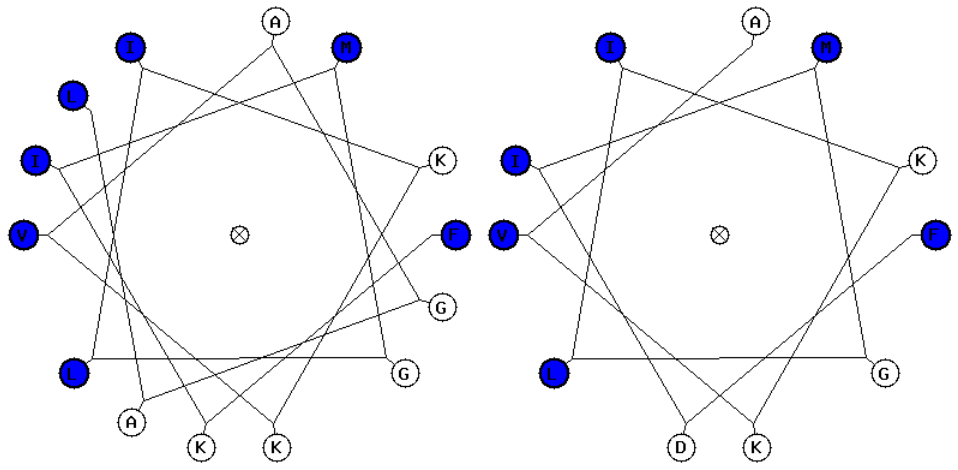

EMP-ER-D2K2 EMP-ER-C3

Figure S1. Schiffer and Edmunson alpha helix wheel projection of EMP-AF and EMP-ER peptides in this work (Software from Helixator: http://www.tcdb.org/progs/helical_wheel.php)

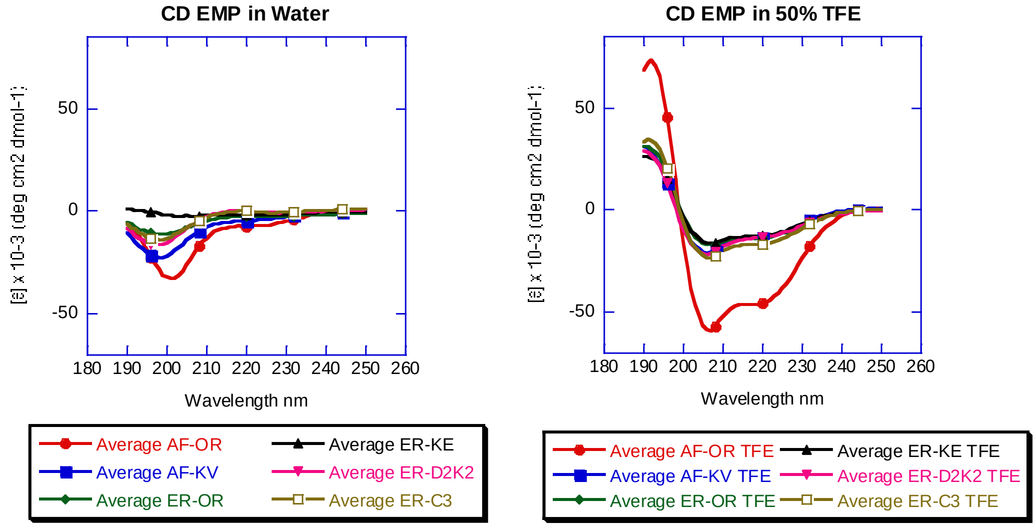

Figure S2. Circular Dichroism plots for secondary structure of EMP peptides:

Table S1. α-Helical Content of EMP Peptides in Water and 50% TFE in Water with errors

Peptides	Water		50% TFE	
	$[\theta]_{222}$	% helix	$[\theta]_{222}$	% helix
EMP-AF-OR	-7711.94±133.91	17.73±0.31	-47630.55±278.05	100±0.87
EMP-AF-KV	-4719.43±45.90	7.85±0.08	-13692.93±190.94	37.47±0.52
EMP-ER-OR	-2759.13±121.41	1.38±0.06	-13698.05±80.71	37.49±0.22
EMP-ER-KE	-2256.20±211.55	Random	-12749.65±63.58	34.35±0.17
EMP-ER-D2K2	-240.03±126.74	6.93±3.66	-13694.72±120.02	37.47±0.33
EMP-ER-C3	-109.85±73.23	7.36±4.91	-18019.75±268.64	51.75±0.77

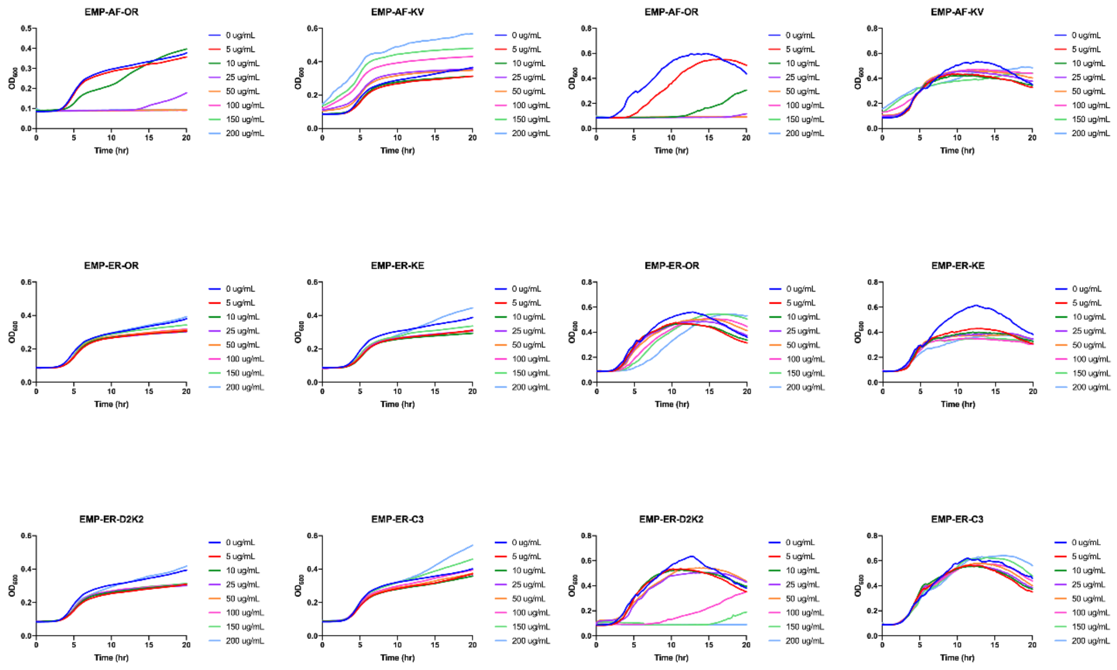

S.aureus ***B. Subtilis***

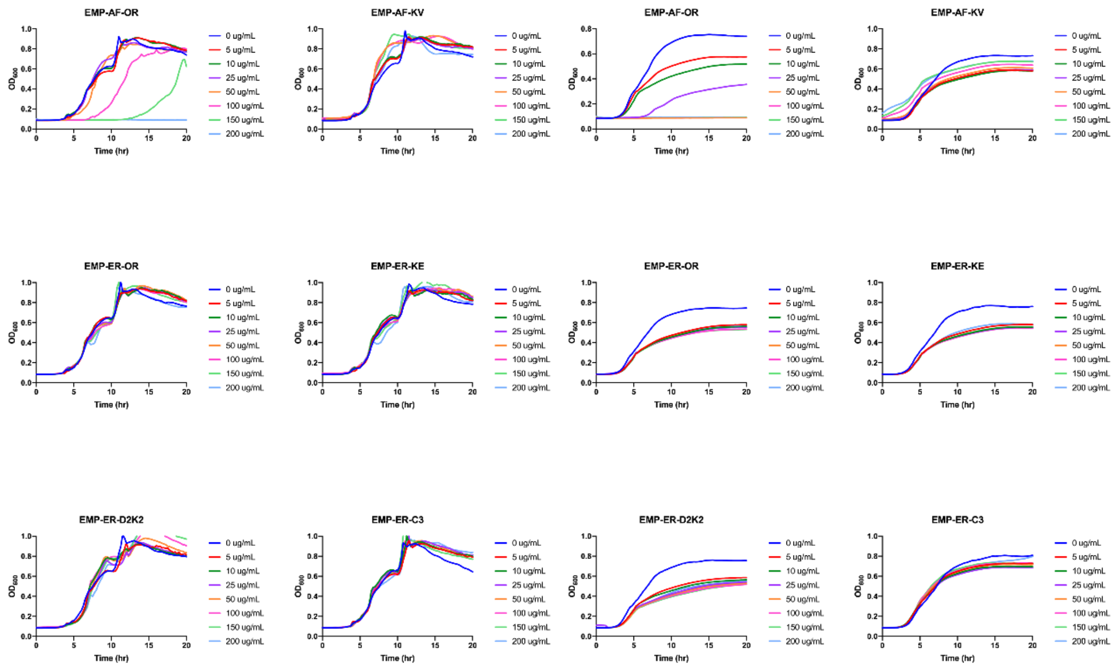

P. aeruginosa *E. coli*

Figure S3. Antibacterial Activity experimental results: plots.

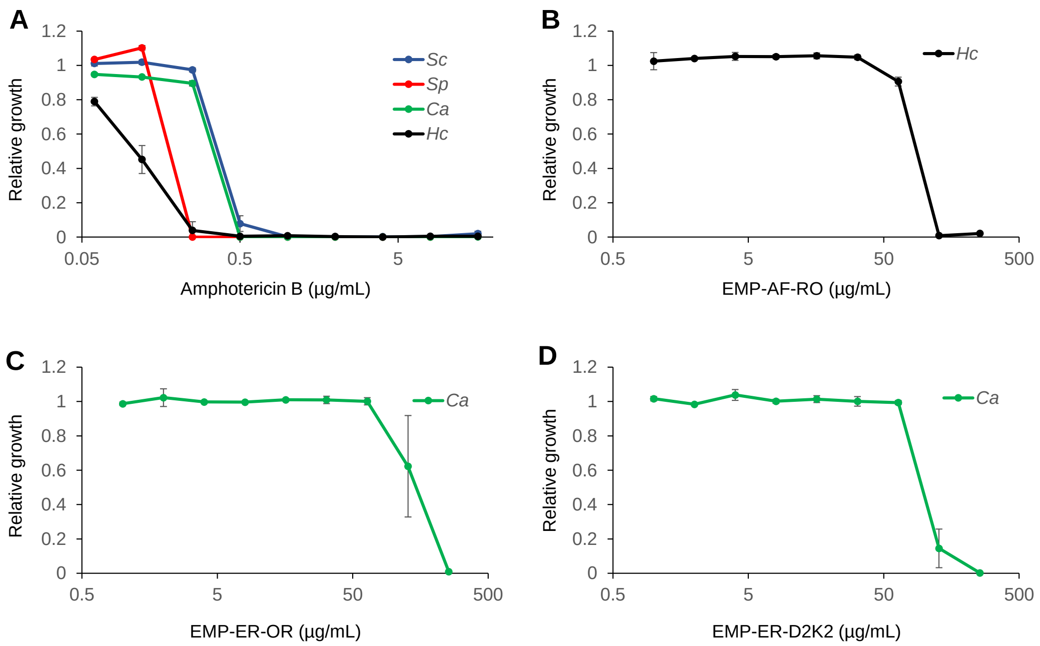

Figure S4. Antifungal dose-response curves tested with 96-well microplate growth assay. Dose-response curves of different fungi (*Sc*: *Saccharomyces cerevisiae* S288C, *Sp*: *Schizosaccharomyces pombe* 972h-, *Ca*: *Candida albicans* SC5314, and *Hc*: *Histoplasma capsulatum* G217B) to amphotericin B **(A)**, EMP-AF-RO **(B)**, EMP-ER-OR **(C)**, and EMP-ER-D2K2 **(D)** was determined by quantitative growth assay (optical density at 595 nm). *S. cerevisiae*, *S. pombe*, and *C. albicans* were inoculated into yeast peptone dextrose (YPD) medium at a density of 1.0×10^4 yeasts/mL and incubated for 24 hours. *H. capsulatum* was inoculated into *Histoplasma*-macrophage medium (HMM) at a density of 1.0×10^6 yeasts/mL and incubated for 96 hours. Fungal growth was measured by optical density 595nm in the end of incubation. Fungal growth was normalized to wells with no peptides or amphotericin B added, and the relative growth was plotted. Data points represent the average ± standard deviation of replicate cultures (n = 3) for each fungal organism tested.
